# Sequence of the *RAG1* and *RAG2* Intergenic Region in Zebrafish (*Danio rerio*)

**DOI:** 10.1155/1998/96749

**Published:** 1998

**Authors:** F. E. Bertrand III, S. L. Olson, C. E. Willett, G. E. Wu

**Affiliations:** 1 Wellesley Hospital Research Institute and the Department of Immunology University of Toronto Toronto Ontario Canada; 2 Department of Biology Massachusetts Institute of Technology Cambridge Massachusetts USA

**Keywords:** RAG, Enhancer, Zebrafish, Lymphocyte, Regulation, Development


*Developmental Immunology*, 1998, Vol. 5, pp. 211–214
Reprints available directly from the publisher
Photocopying permitted by license only

© 1998 OPA (Overseas Publishers Association)
Amsterdam B.V. Published under license
under the Harwood Academic Publishers imprint,
part of the Gordon and Breach Publishing Group
Printed in Malaysia

### *Short Communication*

### Sequence of the *RAG1* and *RAG2* Intergenic Region in Zebrafish (*Danio rerio*)

F. E. BERTRAND[a] III*, S. L. OLSON[a], C. E. WILLETT[b] and G. E. WU[a]

[a]*Wellesley Hospital Research Institute and the Department of Immunology, University of Toronto, Toronto, Ontario, Canada;* [b]*Department of Biology, Massachusetts Institute of Technology, Cambridge, Massachusetts*

*(Received 9 December 1996; In final form 10 January 1997)*

*Keywords:* RAG, Enhancer, Zebrafish, Lymphocyte, Regulation, Development

The recombination activating genes, *rag1* and *rag2* are essential for the rearrangement of antigen receptor V, D, and J gene segments (Oettinger et al., 1990, Mombaerts et al., 1992; Schatz and Oettinger, 1992; Shinkai et al., 1992). Both genes are found in all species that are known to rearrange their antigen-specific receptors. The coding regions as well as the genomic organization of the *rag* locus are highly conserved throughout evolution. *Rag1* and *rag2*, which are convergently transcribed, are separated by an intergenic region of DNA that varies in size among species, being, for example, about 11 kb in the human (*Homo sapiens*), 8 kb in the mouse (*Mus musculus*), 5.2 kb in the frog (*Xenopus laevis*), 2.8 kb in the rainbow trout (*Oncorhynchus mykiss*) (Oettinger et al., 1990; Ichicara et al., 1992; Greenhalgh et al., 1993; Greenhalgh and Steiner., 1995; Hansen and Kaattari, 1996), and 2.6 kb in the zebrafish (*Danio rerio*).

Although *rag1* and *rag2* have been intensively studied, little is known about specific transcriptional control mechanism(s) that regulate their expression. In general, they are coordinately expressed. Only in the chicken bursa, the mammalian brain, and in *Xenopus* oocytes is the expression of one gene found without the other (Carlson et al., 1991; Chun et al., 1991; Greenhalgh et al., 1993). The coordinate expression of the *rag* genes may be mediated by common regulatory elements located in the 3′ intergenic region. Dobbeling and colleagues (1996) have recently provided evidence that elements regulating transcription of the murine *rag* locus may be contained within the 3′ intergenic region. 3′ regulatory elements are known to contribute to the regulation of several lymphoid specific genes, including the immunoglobulin and TCR genes (reviewed in Staudt and Lenardo, 1991; Leiden, 1993). Many of these regulatory elements are conserved among distantly

*Corresponding author. Present address: Wellesley Hospital Research Institute, 160 Wellesley St. E. Toronto, ON M4Y IJ3 Canada.

related vertebrates (Magor et al., 1994). Because the *rag* intergenic region of teleosts is particularly small (Hansen and Kaattari, 1996; Willett et al., 1997), we sequenced this region from the zebrafish and analyzed the sequence for DNA elements likely to govern *rag* expression.

The zebrafish intergenic region is part of a 6.3-kb Sac I fragment of the zebrafish *rag* locus, cloned into pBluescript, as previously described (Willett et al., 1997). A 2.9-kb section of this clone, from the 3′ portion of *rag1* through the 3′ portion of *rag2*, was sequenced on a Pharmacia A.L.F. automatic sequencer (HSC Biotech. Centre, University of Toronto). Sequences were assembled with DNA Strider. The zebrafish *rag* intergenic region of 2,625 bp (Genbank accession U69610) is shown schematically in Figure 1. This region contains no open reading frames longer than 65 amino-acid residues.

Although the nucleotide sequence of enhancers in vertebrates may not be well conserved, studies with the immunoglobulin heavy-chain enhancer in the channel catfish (*Ictalurus punctatus*) and in the trout suggest that the function and regulatory mechanism(s) acting on enhancers are retained throughout vertebrate evolution. The catfish heavy-chain enhancer functions in a B-cell-specific fashion in murine lymphocyte cell lines (Magor et al., 1994). Conversely, the murine heavy-chain enhancer is functional as a transgene in the trout (Michard-Vanhee et al., 1994). Figure 2 summarizes some of the potential regulatory motifs identified in this region by GCG software. Among the enhancer-associated sites identified are motifs for Cu E4.1, Cu E3.1, E2A, and Ets-1 (see Staudt and Lenardo, 1991, for references), which, as in the catfish (Magor et al., 1994), are dispersed over a region, of over 2 kb.

There is a cAMP response element in the *rag* intergenic region at bp 1951. In mammalian species, increases in cAMP results in an increase of *rag1* and *rag2* mRNA, indicating that *rag* expression may be regulated, in part, by cAMP-dependent signaling pathways (Menteski and Gellert, 1990; Bertrand and Wu, unpublished observations). Finding this element in the intergenic region suggests that at least some regulatory activities of the *rag* locus may be attributed to this region.

It has recently been reported that in the trout *rag1* and *rag2* share overlapping 3′ untranslated regions (UTR) (Hansen and Kaattari, 1996). In the zebrafish, the *rag1* and *rag2* 3′ UTR's are approximately 2.3 and 0.8 kb, respectively, assuming a 5′ UTR of about 100 base pairs (Willett et al., 1997). Thus, these transcripts are likely to overlap through the intergenic region. The intergenic region contains multiple potential polyadenylation sites for both *rag1* and *rag2*. The presence of overlapping transcripts and multiple polyadenylation sites in both trout and zebrafish increases the likelihood that expression of *rag1* and *rag2*, in teleosts may involve posttranscriptional regulation through antisense RNA signals (Hansen and Kaattari, 1996).

A striking feature of the RAG intergenic region is a ca. 100 base pair region (1409 to 1506; see Figure 1) with 91% homology to a zebrafish DANA m-1 SINE

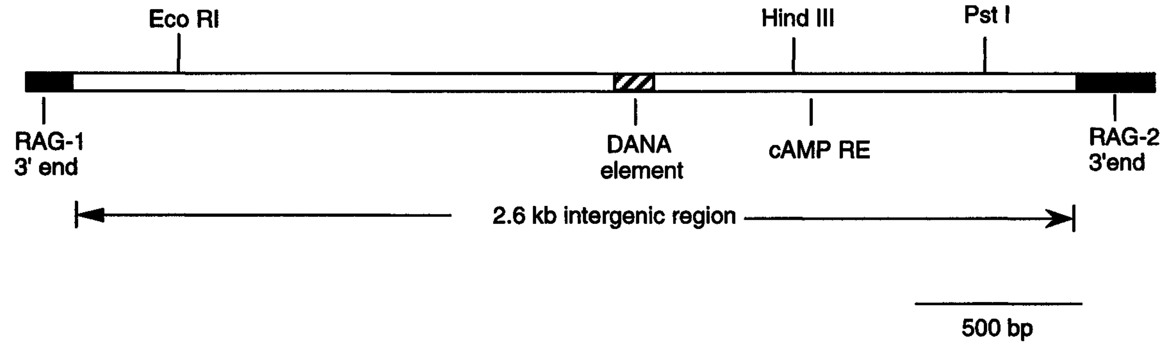

FIGURE 1 Map of zebrafish *rag* intergenic region, indicating the end of the 3′ coding regions of *rag1* and *rag2*, the 2.6-kb region of the zebrafish *rag* intergenic region, and the location of the DANA SINE element. Also shown is the cAMP response element (cAMP RE).

(Izsvak et al., 1995), found by searching the non-redundant (NR) nucleic acid database with the NCBI BLAST server. This sequence falls within the C3-V3-C4-V4 region of the DANA element. Similar sequences are found in the *ependymin, MHC-II,* and the *no-tail* genes of the zebrafish (Izsvak et al., 1996, and references therin). As DANA elements are common in the zebrafish genome, the presence of this remnant in the *rag* locus is most likely fortuitous. However, in light of hypothesises suggesting that the *rag* genes originated from a transposition event (Sakano et al., 1979; Hood et al., 1983; Davis and Bjorkman, 1988; Oettinger et al., 1990; Thompson, 1995), finding transposon-related sequences in the *rag* intergenic region is intriguing.

### *Acknowledgments*

We thank Drs. Charley Steinberg and Lisa Steiner for helpful discussion and critical review of the manuscript. This work was supported by grants from the

| Motif | Sequence | 5' end |
|---|---|---|
| mu-E5/Rev | CAGGTtTT | 191 |
| Ets-1/Rev | ACTTgCTG | 243 |
| NFkB | GGGATTTTTt | 268 |
| CuE4.1 | CAGGTtGT | 283 |
| Ets-1 | CAGGtTGT | 283 |
| CuE5/Rev | ACACCTtCA | 292 |
| CuE4.1 | CAGGaGGT | 450 |
| Ets-1 | CAGGAgGT | 450 |
| mu-E5/Rev | CAGGgGTT | 468 |
| NFkB/Rev | GTAGATCaCC | 568 |
| E-alpha-H-box /Rev | CtGGTCC | 599 |
| E2A | aGGGAATT | 609 |
| NFkB | GGGAATTTTg | 610 |
| Cu4.1/Rev | AcCACCTG | 693 |
| mu-E5 | AACACCTG | 693 |
| CuE5/Rev | AACCTGCt | 694 |
| E2A/Rev | CACCTGc | 695 |
| E2A | TGGtAATT | 883 |
| topo II/Rev | CTCATAATTATAAAC | 1,011 |
| E-box/Rev | GCtACCTG | 1,064 |
| Ets-1/Rev | GCTaCCTG | 1,064 |
| CuE2.1/Rev | GaCAGCTG | 1,290 |
| E2A | ACAGCTG | 1,291 |
| CuE2.1 | CAGCTGGa | 1,292 |
| Ets-1 | GAGGcAGT | 1,397 |
| CuE1.1/Rev | GCCAaCTT | 1,485 |
| Ets-1 | CAGcAAGT | 1,496 |
| Ets-1 | GTGGATGc | 1,512 |
| Ets-1/Rev | ACATCCcC | 1,881 |
| NFkB/Rev | aTAAATCCCC | 1,903 |
| E2A | AATcCCCA | 1,906 |
| Ets-1 | GCtGATGT | 1,949 |
| cAMP RE | TGAtGTCA | 1,951 |
| CuE4.1/Rev | AgCACCTG | 2.087 |
| mu-E5 | AgCACCTG | 2,087 |
| E-alpha-H-box | GcACCTG | 2,088 |
| E2A | CACCTGC | 2,089 |
| mu-E5/Rev | CAGaTGTT | 2,098 |
| CuE4.1/Rev | AtCACCTG | 2,137 |
| E2A | TGaGAATT | 2,188 |
| CuE3.1/Rev | tCCACATG | 2,209 |
| E-alpha-H-Box/Rev | CAGaTCC | 2,247 |
| E2A | CAGCTGC | 2,365 |
| Ets-1/Rev | CCGtAAGT | 2,374 |
| CuE3.1 | CATGTGGa | 2,400 |
| Ets-1 | GTGGATGc | 2,403 |
| Ets-1/Rev | ACATCCTa | 2,464 |
| E2A | AATgCCCA | 2,491 |

FIGURE 2 Selected enhancer motifs (Gosh, 1990) found in the zebrafish RAG locus intergenic region. Small letters indicate base pair mismatch with consensus sequence, and/Rev indicates the reverse of the consensus sequence was found. Base-pair numbering refers to Genbank accession number U69610.

Medical Research Council and National Cancer Institute of Canada, Terry Fox Marathon of Hope. The nucleotide sequence data reported in this paper have been submitted to GenBank and assigned the accession number U6910. C.E.W. is supported by NIH grants 2R01 AI08054, 5T32 AI07436 and 1F32 AI09072.

### References

Carlson L. M., Oettinger M. A., Schatz D. G., Masteller E. L., Hurley E. A., McCormack W. T., Baltimore D. and Thompson C. B. (1991). Selective expression of RAG-2 in chicken B cells undergoing immunoglobulin gene conversion. *Cell*, **64**, 201-208.

Chun J. J., Schatz D. G., Oettinger M. A., Jaenisch R. and Baltimore D. (1991). The recombination activating gene-1 (RAG-1) transcript is present in the murine central nervous system. *Cell*, **64**, 189-200.

Davis M. M. and Bjorkman P. J. (1988). T-cell antigen receptor genes and T-cell recognition. *Nature*, **334**, 395-402.

Dobbeling U., Hobi R., Berchtold M. W. and Kuenzle C. C. (1996). V(D)J recombination is regulated similarly in RAG-transfected fibroblasts and pre-B cells. *J. Mol. Biol.*, **261**, 309-414.

Gosh D. (1990). A relational database of transcription factors. *Nucleic Acids Res.*, **18**, 1749.

Greenhalgh P., Olesen C. E. M. and Steiner L. A. (1993). Characterization and expression of recombination activating genes (RAG-1 and RAG-2) in *Xenopus laevis*. *J. Immunol.*, **151**, 3100-3110.

Greenhalgh P. and Steiner L. A. (1995). Recombination activating gene 1 (RAG 1). in zebrafish and shark. *Immunogenetics*, **41**, 54-55.

Hansen J. D. and Kaatari S. L. (1996). The recombination activating gene 2 (RAG2) of the rainbow trout *Oncorhynchus mykiss*. *Immunogenetics*, **44**, 203-221.

Hood L. E., Hunkapiller T. and Kraig E. (1983). Modern Cell Biology, MacIntosh J. R., Ed. (New York: Liss), 305-328.

Izsvak Z., Ivics Z., Garcai-Estenfania D., Fahrenkrug S. C. and Hakett P. B. (1996). DANA elements: A family of composite, tRNA-derived short interspersed DNA elements associated with mutational activities in zebrafish *(Danio rerio)*. *Proc. Natl. Acad. Sci. USA*, **93**, 1077-1081.

Izsvak Z., Ivics A. and Hackett P. B. (1995). Characterization of a Tc1-like transposable element in zebrafish *(Danio rerio)*. *Mol. Gen. Genet.*, **247**, 312-322.

Leiden J. M. (1993). Transcriptional regulation of T cell receptor genes. *Annu. Rev. Immunol.*, **11**, 539-570.

Magor B. G., Wilson M. R., Miller N. W., Clem L. W., Middleton D. L. and Warr G. W. (1994). An Ig heavy chain enhancer of the channel catfish *Ictalurus punctatus*: Evolutionary conservation of function not structure. *J. Immunol.*, **153**, 5556-5563.

Menteski J. P. and Gellert M. (1990). V(D)J recombination activity in lymphoid lines is increased by agents that elevate cAMP. *Proc. Natl. Acad. Sci. USA*, **87**, 9324-9328.

Michard-Vanhee C., Chourrout D., Stomberg S., Thuvander A. and Pilstrom L. (1994). Lymphocyte expression in transgenic trout by mouse immunoglobulin promoter/enhancer. *Immunogenetics*, **40**, 1-8.

Mombaerts P., Iacomini J., Johnson R. S., Herrup K. and Tonegawa S. (1992) RAG-1 deficient mice have no T and B lymphocytes. *Cell*, **68**, 869-877.

Oettinger M. A., Schatz D. G., Gorka C. and Baltimore D. (1990). RAG-1 and RAG-2, adjacent genes that synergistically activate V(D)J recombination. *Science*, **248**, 1517-1523.

Sakano H., Huppi K., Heinrich G. and Tonegawa S. (1979). Sequences at the somatic recombination sites of immunoglobulin light-chain genes. *Nature*, **280**, 228-294.

Schatz D. G. and Oettinger M. A. (1992). Recombination: Molecular biology and regulation. *Annu. Rev. Immunol.*, **10**, 359-383.

Shinkai Y., Ruthbun G., Lam K. P., Oltz E. M., Stewer T. U., Mendelson M., Charron J., Datta M., Young F., Stall A. M. and Alt F. M. (1992). RAG-2 deficient mice lack lymphocytes owing to inability to initiate V(D)J rearrangement. *Cell*, **68**, 855-867.

Staudt L. M. and Lenardo M. J. (1991). Immunoglobulin gene transcription. *Annu. Rev. Immunol.*, **9**, 373-398.

Thompson C. B. (1995). New insights into V(D)J recombination and its role in the evolution of the immune system. *Immunity*, **3**, 531-539.

Willett C. E., Cherry J. J. and Steiner L. A. (1997). Characterization and expression of the recombination activating genes (*rag1* and *rag2*) of Zebrafish. *Immunogenetics* **45**, 394–404.

